# Evaluation du statut vaccinal et des rappels vaccinaux chez les adolescents scolarisés à Libreville, au Gabon

**DOI:** 10.11604/pamj.2020.35.74.20024

**Published:** 2020-03-16

**Authors:** Ulrick Bisvigou, Eliane Kuissi Kamgaing, Steeve Minto'o Rogombe, Brigitte Adjaou, Euloge Ibinga, Simon Ategbo, Edgard Brice Ngoungou

**Affiliations:** 1 Département d'Epidémiologie, Biostatistiques et Informatique Médicale, Santé Publique, Médecine du Travail et Médecine Légale, Faculté de Médecine, Université des Sciences de la Santé, Libreville-Owendo, Gabon; 2 Unité de Recherche en Epidémiologie des Maladies Chroniques et Santé Environnement, Faculté de Médecine, Université des Sciences de la Santé, Libreville-Owendo, Gabon; 3 Département de Pédiatrie, Faculté de Médecine, Université des sciences de la Santé, Libreville-Owendo, Gabon; 4 Service de Néonatalogie, Centre Hospitalo-Universitaire Fondation Jeanne Ebori, Libreville, Gabon; 5 Service de Pédiatrie, Centre Hospitalo-Universitaire Fondation Jeanne Ebori, Libreville, Gabon

**Keywords:** Adolescent, calendrier vaccinal, Gabon, Programme Elargi de Vaccination, système éducatif, Adolescent, immunization schedule, Gabon, Expanded Program of Immunization, educational system

## Abstract

**Introduction:**

La vaccination chez l'adolescent est particulière et le statut vaccinal de ce dernier est peu connu. L'objectif de cette étude était d'apprécier ce statut vaccinal et d'identifier les facteurs associés à la compliance vaccinale chez les adolescents scolarisés à Libreville.

**Méthodes:**

Une enquête observationnelle transversale descriptive a été réalisée chez les élèves des classes de 6^e^du Lycée national Léon MBA de Libreville.

**Résultats:**

Au total, 304 élèves ont été inclus dans l'étude. L'âge moyen des élèves était de 11,60±1,20 ans et le sexe ratio était de 0,6. Deux cent soixante-six enfants (87,5%) vivaient avec leur géniteur direct (père et/ou mère). Le nombre moyen d'enfants par famille était de 4. Le taux de couverture vaccinale était de 78,3%. Le nombre d'enfants par famille n'était pas associée à la couverture vaccinale des vaccins du PEV (p=0,088), par contre les enfants vivant avec au moins l'un des parents avaient une meilleure couverture vaccinale respectivement par les vaccins du PEV (p=0,025) et les vaccins hors PEV (p=0,035). Les facteurs évoqués par les parents pour expliquer la non-vaccination étaient le manque d'information (30,59%), l'oubli (24,67%) et le manque de moyens financiers (12,82%).

**Conclusion:**

La couverture vaccinale des adolescents scolarisés à Libreville semble relativement proche des objectifs du PEV, mais elle reste associée à la situation familiale. D'autres campagnes de sensibilisation seraient utiles pour améliorer cette couverture vaccinale au Gabon.

## Abstract (en)

**Introduction:**

Vaccination is targeted to selected adolescents and their vaccination status is little known. The purpose of this study is to assess the vaccination status and to identify factors associated with vaccination compliance in adolescents attending school in Libreville.

**Methods:**

A cross sectional observational descriptive survey was conducted among students attending grades 6 at the Leon Mba high school in Libreville.

**Results:**

In total, 304 students were included in the study. The average age of students was 11.60±1.20 years and sex ratio was 0.6. Two hundred and sixty-six children (87.5%) lived with their parent (father and/or mother). The average number of children per family was 4. Immunization coverage rate was 78.3%. The number of children per family was not associated with vaccine coverage of Expanded Program on Immunization (EPI) vaccines (p=0.088) while children living with at least one of their parents had a better immunization coverage for EPI vaccines (p=0.025) and vaccines outside EPI (p=0.035) respectively. Factors reported by parents to explain reasons for non-vaccination were the lack of information (30.59%), forgetfulness (24.67%) and the lack of financial resources (12.82%).

**Conclusion:**

Vaccination coverage for adolescents attending school in Libreville appears to be relatively close to the goals of EPI, but it is still associated with family situation. Other awareness campaigns would be useful to improve vaccination coverage in Gabon.

## Introduction

L'adolescence, période comprise entre l'âge de 10 et 19 ans, est une période de transition critique en matière de santé. Selon le rapport de l'Organisation Mondiale de la Santé (OMS), sur la situation de la population mondiale, la proportion des adolescents et des jeunes vivant en Afrique devrait augmenter et passer de 18% en 2014 à 30% en 2050 [1]. Le nombre d'adolescent est estimé à environ 1,2 milliard de personnes, soit un sixième de la population mondiale. Du point de vue sanitaire cette période, qui succède à la petite enfance, objet de nombreux programmes nationaux de lutte contre la mortalité et la morbidité, fait très peu l'objet de programme de santé. Pourtant les adolescents peuvent aussi contracter ou développer des affections telles que les traumatismes, l'obésité, les addictions et les maladies infectieuses qui auront un impact considérable sur la qualité de leur vie d'adulte ou celle de leurs futurs enfants [1,2]. Certaines de ces maladies sont des maladies infectieuses pouvant être prévenues par la vaccination. Cependant, celle-ci reste particulière chez l'adolescent du fait qu'elle doit non seulement permettre de consolider l'immunité acquise par les vaccins de la petite enfance grâce aux rappels, mais doit aussi intégrer d'autres vaccins liés à d'autres maladies infectieuses auxquelles est désormais exposé l'adolescent, du fait de son comportement. C'est le cas des infections à *Human papilloma virus* (HPV) et des hépatites (A et B). La vaccination représente l'un des plus beaux succès de la santé publique au 20^e^ siècle. Elle a permis, à un moindre coût, de sauver des millions de vies d'enfants à travers les Programmes Elargi de Vaccination (PEV) ou Programmes Nationaux de Vaccination, et avec l'aide des organisations internationales partenaires des Etats telles que l'UNICEF (Fonds des Nations Unies pour l'Enfance) ou GAVI Alliance (*Global Alliance for Vaccin and Immunisation*) [3]. Selon les estimations de l'OMS, la vaccination a prévenu environ 2,9 millions de décès par la rougeole, le tétanos néonatal, la coqueluche et la poliomyélite en 2002 [4]. Cependant, le bénéfice apporté par la vaccination dans l'enfance s'estompe, au fil du temps en l'absence de rappels vaccinaux [5-7]. Au Gabon, à l'instar de nombreux pays de l'Afrique subsaharienne, il existe un Programme Elargi de Vaccination. Lancé en 1978, il n'a été effectif que depuis 1990. N'étant pas éligible par le GAVI Alliance, le Gabon a pu déployer et soutenir son PEV sur le territoire. Il existe actuellement 119 centres fixes fonctionnels de vaccination, équipés et intégrés dans les établissements sanitaires au Gabon. Ils y réalisent la vaccination de routine concernant les vaccins contre la tuberculose avec le Bacille de Calmet et Guérin (BCG), la poliomyélite avec le vaccin polio oral et injectable (VPO, VPI), la diphtérie, le tétanos et la coqueluche (DTC), l'hépatite B et l'*Haemophilus influenzae*, la rougeole (VAR) et la fièvre jaune (VAA). Ces vaccins sont administrés sur un calendrier comprenant 10 rendez-vous associés aux âges de l'enfant à vacciner: naissance, 6 semaines, 10 semaines, 14 semaines et 9 mois. Du fait que les vaccins du PEV au Gabon soient gratuits jusqu'à l'âge de 11 mois, la couverture vaccinale des enfants de moins d'un an, et même parfois jusqu'à cinq ans, est mieux connue à travers les chiffres officiels et les enquêtes sur la couverture vaccinale [8]. Par contre, très peu de données existent sur l'effectivité de la vaccination et des rappels vaccinaux chez l'adolescent. Au vu de ce constat, il nous a semblé opportun de connaître le taux de couverture vaccinale et les facteurs potentiels associés à la vaccination des enfants scolarisés de cette catégorie d'âge au Gabon. L'objectif de cette étude a été d'évaluer le taux de couverture vaccinale et l'effectivité des rappels vaccinaux, selon le PEV et les vaccins hors PEV des adolescents scolarisés dans un établissement public au Gabon.

## Méthodes

Il s'agissait d'une enquête observationnelle de type transversale à visée descriptive et analytique effectuée dans un établissement scolaire public secondaire de Libreville. Celle-ci a été réalisée du 1^er^ avril au 15 juin 2015 au Lycée National Léon MBA (LNLM). Cet établissement secondaire est l'un des plus grands et anciens établissements publiques du Gabon. Il est situé dans la circonscription scolaire nord de Libreville et compte tous les niveaux de l'enseignement secondaire général. L'ensemble des élèves inscrits en classe de 6^e^ constituaient la population source de cette étude. Etaient inclus tous les élèves du LNLM inscrits en classe sixième, présents lors du passage des enquêteurs et dont les parents avaient donné leur consentement. L'étude s'est déroulée en deux phases. La première phase a consisté à contacter les parents, à l'aide d'une fiche d'information et de sollicitation de consentement, et à sélectionner les élèves en fonction de la présence ou non d'un carnet de vaccination. La deuxième phase a consisté à examiner de manière approfondie le carnet de vaccination, à relever les données inhérentes à chaque enfant et la situation sociodémographique et professionnelle des parents/tuteurs. L'examen du carnet portait entre autres sur les différents antigènes ainsi que leurs dates d'administration, l'âge de l'élève au moment de l'administration de chaque vaccin ou antigène et en fonction des rendez-vous du calendrier vaccinal en vigueur au Gabon permettant de mettre en évidence une avance, une adéquation (à temps) ou un retard. Les rappels vaccinaux ont été également étudiés de la même manière, essentiellement, les vaccins contre la rougeole-oreillon-rubéole (ROR), les méningocoques A+C, la fièvre typhoïde et le pneumocoque (Pneumo23). L'ensemble des informations ont été reportées sur une fiche de recueil de données standardisée. Les données ont été analysées avec le logiciel Epi InfoTM version 7.0.8.3. Les données qualitatives ont été décrites en pourcentage et comparées à l'aide du test de Chi-2 Pearson et/ou test exact de Fisher (pour les effectifs attendus inférieurs à 5); les données quantitatives ont été décrites en moyenne ± Ecart-type ou en médiane (minimum et maximum), et la comparaison des moyennes a été faite avec le test de t Student. Le seuil de significativité admis était α=0,05.

**Considérations éthiques**: cette étude a obtenu l'autorisation du Ministère de l'Education nationale et du Ministère de la Santé du Gabon ainsi que des autorités du Lycée national Léon Mba. Un consentement éclairé des parents/tuteurs était requis pour chaque participant.

## Résultats

Neuf cent quatre-vingt-dix élèves étaient inscrits en classe de 6^e^au LNLM, au cours de l'année scolaire 2015-2016. Parmi lesquels, 686 n'ont pas été inclus dont 554 pour absence de carnet de vaccination et 132 pour refus des parents. Au total, 304 élèves ont été inclus.

**Caractères sociodémographiques des élèves**: les élèves étaient âgés de 10 à 16 ans avec une moyenne d'âge de 11,6±1,2 ans. Le sexe ratio était de 0,67. Le nombre d'enfant par famille allait de 1 à 18, avec une moyenne de 4,3±2,1 enfants. Le rang occupé par les élèves dans la fratrie, allait de premier à dixième dans la fratrie, le rang médian était le deuxième. L'âge des mères allaient de 25 à 59 ans avec une moyenne de 38,9±6,4 ans. Quatre-vingt pour cent des élèves étaient de nationalité gabonaise. Deux cent soixante-six élèves (87,5%) vivaient avec au moins un de leur géniteur direct (père et/ou mère). Le Tableau 1 décrit les principales variables sociodémographiques explorées. Les principaux résultats sur les doses ou antigènes reçus par les élèves sont contenus dans le Tableau 2. A leur naissance, 40 élèves (13,15%) n'ont pas reçu de dose de BCG. Vingt-sept élèves (8,88%) étaient en retard par rapport à la première dose de VPO (VPO0), administrée à la naissance, 59 (19,41%) ne l'avaient pas reçu à la naissance. Pour la dose de VPO requise à 6 semaines, 245 élèves (80,69%) l'avaient reçu. Par rapport au premier rendez-vous de vaccination, 264 élèves (86,84%) étaient à jour, 4 élèves étaient en avances sur leur vaccination. Seulement, 172 élèves (56,57%) ont été vaccinés à temps contre la rougeole. Chez ces élèves, environ 118 (38,80%) avaient reçu la première dose de VAA, 3 mois à 1 an après la date prévue. La couverture vaccinale PEV (en associant DTC3, Fièvre jaune et rougeole) est de 54,93% (167/304). Pour ces 167 adolescents, les taux pour les premiers, deuxièmes et troisièmes rappels de DTP étaient respectivement de 80,23% (134/167), 41,9% (70/167) et 29,34% (49/167). On avait aussi 8,38% (14/167) pour la fièvre jaune, 4,19% (7/167) pour la rougeole et ROR2 et 25,74% (43/167) pour l'hépatite B.

**Tableau 1:** Répartition des caractéristiques sociodémographiques des familles de 304 élèves de classe de 6^e^ enquêtés au Lycée national Léon Mba de Libreville en 2015

Variable	Fréquence	Pourcentage (%)
**Activité de la mère**		
Femme au foyer	95	(31,2)
Femme active	197	(64,8)
Etudiante/en formation	12	(3,9)
**Niveau d’étude de la mère**		
Primaire	51	(19,1)
Secondaire	147	(55,0)
Universitaire	52	(19,4)
Non scolarisée	17	(6,3)
**Type de famille**		
Monoparentale	90	(29,6)
Couple	214	(70,4)
**Profession du chef de famille**		
Sans emploi	68	(22,3)
Cadre secteur publique/privé	90	(29,8)
Non cadre secteur privé	41	(13,4)
Non cadre secteur publique	46	(15,1)
Travailleur indépendant	59	(19,4)
**Revenu mensuel du chef de famille**		
Moins de USD 145	29	(10,8)
Entre USD 145 et USD 364	75	(27,9)
Entre USD 364 et US 910	115	(42,9)
Plus de USD 910	49	(18,2)

**Tableau 2:** Description du statut vaccinal de 304 élèves de classe de 6^e^ du Lycée national Léon Mba de Libreville, en fonction de la classe d’âge et du calendrier du PEV, en 2015

Antigène/Vaccin (Classe d’âge)	A temps		En avance		En retard		Total vacciné	
	n	%	n	%	n	%	n	%
**BCG (naissance)**	264	86,80	0	0,00	0	0,00	264	86,80
**VPO (naissance)**	218	71,70	0	0,00	27	8,80	245	80,50
**VPO1 (6 semaines)**	14	4,60	0	0,00	22	7,10	36	11,80
**Penta 1 (6 semaines)**	254	83,50	4	1,30	0	0,00	258	84,89
**VPO2 (10 semaines)**	11	3,60	0	0,00	18	5,90	29	9,50
**Penta 2 (10 semaines)**	245	80,90	4	1,30	0	0,00	249	81,90
**VPO3 (14 semaines)**	3	0,90	0	0,00	1	0,32	4	1,31
**Penta 3 (14 semaines)**	233	76,60	4	1,30	1	0,32	238	78,28
**VAR (9 mois)**	172	56,50	0	0,00	33	10,8	205	67,43
**VAA (9 mois)**	57	18,70	0	0,00	124	40,77	181	59,52

**Figure 1:**
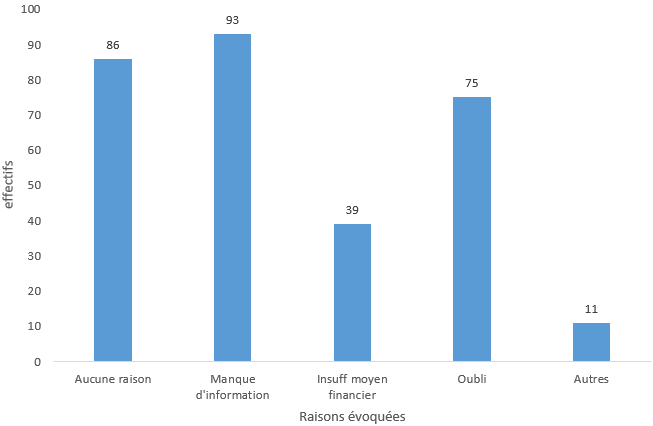
Description des raisons évoquées par les parents pour justifier l'absence de vaccination des élèves de classe de 6^e^ du LNLM de Libreville, en 2015

Les taux de couverture pour les vaccins hors PEV étaient les suivants 24,01% pour la première dose de méningocoque (73/304), avec un taux de rappel de 1,9% (6/304) ; 22,03% pour la première dose de vaccin anti fièvre typhoïde (67/304), le taux de rappels pour la deuxième et la troisième dose étaient respectivement de 2,30% (7/304) et 0,9% (3/304) ; pour la première dose de pneumocoque 15,78% (48/304) et pour la dose de rappel 0,32% (1/304). Seuls deux adolescents avaient reçu une dose de vaccin contre la grippe. Le manque d'information précise était la principale raison évoquée par 129 (43%) parents pour expliquer l'absence de vaccination des élèves. Venaient ensuite le manque de moyens financiers 69 (18%) et l'oubli 92 (34%) (Figure 1).

Globalement, le statut vaccinal des adolescents par rapport au PEV, c'est-à-dire l'administration correcte de la troisième dose de DTC (DTC3) chez les élèves n'était pas associée au lien de parenté avec le chef de famille dont 78,35% vivaient avec leurs parents directs contre 63% avec un autre parent (p=0,142). Une différence significative était observée, pour l'administration du BCG à la naissance, entre les adolescents vivant avec leur parent géniteur (88,43%) et ceux vivant avec un tuteur (75,00%) (p=0,025). Il existait une différence significative, pour l'administration du DTC1 à temps, entre les adolescents issus de familles monoparentales (73,33%) et ceux issus de familles biparentales (87,85%) (p<0,001). Il existait aussi une différence significative des proportions d'adolescents ayant reçu le DTC2 pour une famille monoparentale (72,22%) et une famille biparentale (84,57%) (p=0,006). S'agissant des vaccins hors PEV, la couverture vaccinale était meilleure pour les élèves vivant avec un des parents géniteur que ceux vivant avec un autre parent pour le BCG 20,14% contre 2,77% (p=0,008). La couverture vaccinale était également meilleure pour les enfants vivant avec leurs deux parents pour le vaccin contre la fièvre jaune (VAA), 51,92% contre 27,77% (p=0,003), la méningite 25,74% contre 5,55% (p=0,022) et la fièvre typhoïde 23,88% contre 8,33% (p=0,035). Par rapport à l'effectivité vaccinale, les adolescents dont la raison de non vaccination était le manque de moyen financier avaient une moins bonne couverture vaccinale pour le ROR que ceux dont les parents avaient évoqué une tout autre raison, 7,5% contre 20,8% (p<0,001) ; de même pour la couverture contre la fièvre typhoïde, 6,4% contre 9,6% (p<0,001) (Tableau 3). Les raisons évoquées (manque d'informations, de moyen financier et l'oubli) étaient fortement associées à une mauvaise couverture vaccinale (p<0,001).

**Tableau 3:** Effectivité vaccinale des vaccins PEV et hors PEV, en fonction des raisons évoquées par les parents des élèves de 6^e^ du LNLM, en 2015, pour justifier l’absence de vaccination ou de rappels vaccinaux

Variables	Insuffisance de moyens financiers		Autres raisons*		*p*
	Effectifs (n=93)	%	Effectifs (n=125)	%	
**BCG1**	89	95,6	105	84,0	0,006
**DTCP**	77	82,7	99	79,2	<0,001
**Fièvre typhoïde**	6	6,4	12	9,6	<0,001
**ROR**	7	7,5	26	20,8	<0,001

## Discussion

Nous avons évalué le taux de couverture vaccinale et estimé le taux des rappels vaccinaux des adolescents scolarisés dans un des plus grands établissements secondaires de Libreville. Cette étude a permis de dresser un état des lieux de la vaccination chez les adolescents au Gabon. En effet, ce pays ne bénéficiant pas du soutien du programme GAVI (Global Alliance for Vaccine and Immunisation), il semblait opportun, pour une meilleure prise de décision par les autorités sanitaires, de mesurer l'impact des vaccins PEV et des vaccins hors PEV. Sur le plan des maladies infectieuses, il est important et nécessaire d'évaluer le niveau de protection des adolescents contre les maladies évitables par la vaccination. Cette étude permet aussi d'avoir une idée l'implication des parents dans le suivi des rappels vaccinaux des adolescents.

**Absence de carnet de vaccination**: l'utilisation du carnet de santé pour étudier la couverture vaccinale est courante dans de nombreuses études [9]. Cette méthode est aussi de plus en plus abandonnée au profit de registres électroniques qui donnent des informations, en temps réel, plus proches de la réalité [10,11]. L'absence de carnet de vaccination a été un grand handicap pour cette enquête car il n'a pas été possible de connaitre le statut vaccinal réel de la plupart des élèves du LNLM. Il est admis dans certaines communautés que la vaccination ne concerne que les enfants de moins de 12 mois, c'est-à-dire la période couverte par le PEV, qu'elle ne concerne pas les adultes, encore moins les adolescents. Ce qui expliquerait la négligence envers ce précieux document, qui pourtant permet d'assurer la continuité des soins. Au Gabon, un carnet de santé avec des vaccins à jour est exigé à l'entrée à l'école maternelle, que ce soit dans le secteur privé ou publique. Ce qui oblige les parents à compléter la vaccination de leurs enfants avant leur introduction dans les collectivités scolaires. Ce n'est pas le cas à l'entrée du collège; même si un certificat médical est demandé aux élèves en fin de cycle primaire pour passer le Certificat d'Etudes du Premier Cycle, leur permettant d'accéder à la classe de 6e ; un carnet de vaccination à jour n'est pas exigé lors de cet examen médical. Comme il a été déjà démontré ailleurs, la surveillance des carnets améliore la couverture vaccinale. Au cours des discussions avec les parents, nombre d'entre eux ont reconnu que tant que leur enfant n'était pas malade, leur carnet de santé n'était pas consulté et restait rangé dans un sac au fond d'un placard. Seule la nécessité de consultation du pédiatre ou d'un personnel de santé les amenait à le sortir pour le présenter. Et ce n'était qu'à ce moment que la question de la complétude des vaccins était remise à l'ordre du jour. D'où ici tout l'intérêt, d'intégrer des systèmes de rappel aux parents. Ces systèmes peuvent prendre la forme d'appels téléphoniques ou d'envois de messages (SMS), cette méthode est de plus en plus employée à travers le monde [12-14]. Cette pratique est utilisée dans certains centre de vaccination au Gabon, mais restent toujours rudimentaire et informelle ; le but ici est surtout de limiter les pertes en vaccins et d'appliquer la politique du flacon entamé [15]. Nous n'avons pas, au cours de notre enquête, explorer les raisons qui pouvaient amener les parents à maintenir une certaine vigilance vaccinale. C'est le cas des enfants pour qui la fréquentation des hôpitaux est quasi régulière, comme les drépanocytaires, ou l'exigence pour un voyage international. Car, en plus des vaccins prescrits dans l'enfance, d'autres vaccins, à cause des particularités de l'adolescence, l'entrée dans la sexualité par exemple, doivent faire l'objet de vigilance et de recommandation. C'est le cas pour l'hépatite B ou l'HPV [16].

**Couverture vaccinale PEV**: selon l'OMS on peut considérer qu'un enfant est complètement vacciné s'il a reçu le BCG à la naissance, la troisième dose de DTC, la troisième dose de Polio orale, une dose de VAA (fièvre jaune) et une dose de VAR (rougeole). En considérant cet aspect, la couverture vaccinale au Gabon est alors estimée à 32% sur le territoire gabonais et 36% dans l'estuaire, notre région d'étude en 2013 [17]. Nos lycéens ont une couverture estimée, selon les mêmes critères à 56,93%. Ce qui suggère que la couverture vaccinale est meilleure en milieu scolaire que dans la population générale, estimée à 32% [17]. Ayant un taux d'alphabétisation de 88% pour les 15-24 ans, les établissements scolaires du Gabon peuvent être aussi un moyen de promotion de la vaccination, comme les autres thématiques de promotion de la santé des adolescents.

**La gestion des stocks de vaccin**: en pratique, le BCG et le VPO0 sont administrés le même jour, nous devrions donc avoir les mêmes proportions de vaccinés pour ces deux antigènes, ce qui n'a pas été le cas dans notre série. En discutant avec les acteurs de la vaccination, l'explication serait la survenue inopportune de rupture de stock de l'un ou l'autre des antigènes, au sein du centre de vaccination. L'un des antigènes sera alors administré alors que l'autre ne le sera pas. Les parents ne sont pas souvent vigilants et proactifs, le vaccin non administré ne le sera plus. La révision de la procédure de gestion des stocks de vaccin peut ici aussi être à l'origine d'une mauvaise vaccination comme cela a été observé à Kinsangani [18], ce qui pourrait constituer une autre occasion manquée pour non disponibilité du vaccin.

**Caractère sociodémographiques des élèves**: ni le nombre d'enfant par famille, ni le revenu des familles n'influençaient, dans notre série, la couverture et la complétude vaccinale par rapport au PEV. Du fait que ces vaccins sont pris en charge par l'Etat, seuls les coûts indirects de la vaccination comme le coût du transport pourraient être un handicap pour les familles à faible revenus. D'autres facteurs ici pourraient également intervenir comme l'hésitation à la vaccination mais ce facteur n'a pas été exploré au cours de notre étude.

**Rappels vaccinaux et vaccination hors PEV**: les élèves dont le chef de famille est le père et/ou la mère avaient un meilleur statut vaccinal pour les vaccins hors PEV, même si cette vaccination hors PEV n'avait pas été souvent complétée. Ce qui met encore le parent au centre de la responsabilité de la vaccination de l'enfant. Deux maladies évitables par la vaccination font depuis l'objet d'attention particulière chez les adolescents, hépatite B et l'infection à *Human Papilloma virus* (HPV), responsables des cancers du foie et du col de l'utérus. Pour ces deux infections, il existe des antigènes dont l'administration devrait se faire impérativement au début de l'adolescence, non pris en charge par le PEV. S'il existe pour les vaccins du PEV, une difficulté d'intégration dans les *habitus* parentaux, qu'en serait-il pour d'autres vaccins en cours d'introduction dans les pays en voie de développement où ces maladies sont quasi-endémiques.

**Raisons de non vaccination**: les raisons de non-adhésion à la vaccination au Gabon sont assez connues: éloignement des centres de santé, coût du transport, mauvaise expérience avec le personnel de santé, manque de moyens et raisons liées à la pauvreté [19]. Le manque d'information était le premier ennemi de la vaccination dans notre étude; venait ensuite l'oubli et le manque de moyens financier . Ce dernier était plus lié aux rappels vaccinaux et aux vaccins hors PEV qui sont à la charge des parents. En dehors de ces trois principales, les autres raisons évoquées pour justifier l'absence de vaccination étaient la perte du carnet ou son indisponibilité, le manque de volonté, le manque d'intérêt pour la vaccination. Au regard des raisons évoquées par les parents pour expliquer l'absence ou le retard à la vaccination, le manque de moyen financier n'influençait pas la couverture vaccinale PEV, puisque ces vaccins sont pris en charge, mais détermine les rappels vaccinaux PEV et la vaccination hors PEV. Les adolescents dont la raison de non vaccination était le manque de moyen financier avaient une moins bonne couverture vaccinale pour le ROR que ceux dont les parents avaient évoqué une autre raison, 7,5% contre 20,8% (p<0,001) ; de même pour la couverture contre la fièvre typhoïde, 6,4% contre 9,6% (p<0,001). Les raisons évoquées (manque d'informations, de moyen financier et l'oubli) étaient fortement associées à une mauvaise couverture vaccinale (p<0,001).

**Promotion de la vaccination**: au Gabon, le vaccin contre l'hépatite B a été introduit en 2004 en combinaison à 6, 10 et 14 semaines , ce qui pourrait expliquer sa faible couverture chez les enfants à la naissance étant donné que la dose monovalente des hépatites B n'est réalisée que dans les structures sanitaires privées. Aucun plan médiatique n'avait été prévu pour promouvoir ce vaccin [10]. En général tant que l'enfant n'est pas malade, les parents ne consultent pas régulièrement le carnet de santé de leur enfant. Tant que le pédiatre ou le personnel de santé ne rappel pas aux parents le fait du retard ou du rappel des rendez-vous ou des vaccins les parents ne sont pas sensibles. Globalement le statut vaccinal par rapport au PEV n'a pas de différence quel que soit le parent qui est en charge de l'enfant. Mais on remarque que s'agissant des vaccins hors PEV la couverture est meilleure pour les enfants qui sont chez leurs parents directs. L'état doit ici reprendre son rôle de régalien et profiter du système scolaire pour vacciner tous les enfants. Comment améliorer la couverture vaccinale des adolescents? C'est par la promotion des rappels vaccinaux [20] à travers la prescription des personnels de santé ou l'achat direct en pharmacie [21]. Pour les adolescents ayant des maladies chroniques comme la drépanocytose ou l'épilepsie c'est l'occasion de corriger les parcours de soins. L'adolescent n'était pas il y a une quinzaine la cible de programme national de vaccination, en dehors de l'HPV. Les couvertures vaccinales des antigènes du PEV sont faibles par rapports aux objectifs dans la population adolescente de Libreville. Des efforts doivent être faits pour promouvoir l'intérêt de cette activité auprès des parents, des éducateurs et du système de santé car le Programme national de vaccination ne tient pas compte de cette tranche d'âge. Les adolescents ont besoin d'être protégés contre les maladies courantes mais aussi contre l'hépatite B et l'HPV du fait de l'ouverture à la sexualité. Et le seul budget du PEV ne peut pas l'assurer.

Certains auteurs indiquent que la couverture vaccinale aurait été la plus élevée au Gabon dans les années 1980 car les campagnes de vaccinations menées par le Service de Grandes Endémie couvrait même les écoles [20]. Le PEV du Gabon, sous sa forme actuelle, financé entièrement par l'Etat, sur fond propre via l'UNICEF, ne fait plus de campagnes ciblées vers les établissements scolaires ou les collectivités en dehors des campagnes de riposte à des maladies précises comme la rougeole ou la poliomyélite. Les ressources mobilisées par le gouvernement gabonais et ses partenaires, pour faire vivre ce programme, ne permettent pas d'atteindre les performances annoncées de 90%. Il n'existe pas à notre connaissance des données publiées sur la vaccination des adolescents au Gabon. Les données concernent surtout les enfants de moins de 5 ans qui font l'objet du PEV et des activités de vaccination supplémentaires (campagnes contre la rougeole ou contre la poliomyélite), ce qui est similaire à ce que l'on a pu observer dans d'autres pays [22-24]. De plus, en Afrique subsaharienne, les programmes gouvernementaux de vaccination dédiés aux adolescents sont quasi inexistants, à part ceux développés récemment pour le vaccin contre HPV (*Human Papilloma Virus*). Dans la lutte contre ce virus, en 2006, 9% des pays africains ont pour cible vaccinale les 9-20 ans Algérie, Lybie, Tunisie, Namibie. Enfin, il est possible d'imaginer des passerelles ou check point de suivi médical, parallèle au cursus scolaire, entre l'adolescence sous la responsabilité du pédiatre, et la médecine adulte, comme au Chili par exemple [25]; le système éducatif d'un pays peut mettre en place des mesures pour garantir la santé et l'éducation, en rattrapant et mettant à jour les vaccinations non faites, dans leurs infirmeries respectives, à la veille et lors des grands examens nationaux tels que le Certificat d'Etudes Primaires (CEP), le Brevet d'Etudes du Premier Cycle du second degré (BEPC) ou le Baccalauréat (BAC).

## Conclusion

La couverture vaccinale des élèves en début de collège est certes meilleure que celle de la population générale au Gabon. Les enjeux de la couverture vaccinale des adolescents, ne faisant pas partie du Programme Elargi de Vaccination, ils ne sont pas bien appréhendés ni par les parents ni par le système éducatif. Nous avons pu mettre en évidence la part du système familial dans la prise en compte de la complétude de la vaccination. Une prise de conscience est nécessaire car ces adolescents représentent de futurs adultes dont la proportion est croissante.

### Etat des connaissances actuelles sur le sujet

- La couverture vaccinale globale des enfants vaccinés par le PEV de 1 an jusqu'à 5 ans;
- Manque d'informations sur la couverture vaccinale et les rappels vaccinaux des adolescents;
- Manque d'information sur les facteurs de risque liée à l'absence de vaccination.

### Contribution de notre étude à la connaissance

- Le taux de couverture vaccinale et des rappels vaccinaux des adolescents scolarisés selon le PEV, à Libreville;
- Le taux de couverture vaccinale et des rappels vaccinaux en dehors du PEV à Libreville;
- L'identification de certains facteurs déterminants la couverture vaccinale chez l'adolescent au Gabon.

## Conflits d’intérêts

Les auteurs ne déclarent aucun conflit d'intérêts.
